# Effects of feeding high-energy diet on growth performance, blood parameters, and carcass traits in Hanwoo steers

**DOI:** 10.5713/ab.22.0014

**Published:** 2022-05-02

**Authors:** Dong Hun Kang, Ki Yong Chung, Bo Hye Park, Ui Hyung Kim, Sun Sik Jang, Zachary K. Smith, Jongkyoo Kim

**Affiliations:** 1 Department of Animal Science, Korea National University of Agriculture and Fisheries, Jeonju 54874, Korea; 2 Hanwoo Research Institute, National Institute of Animal Science, Pyeongchang 25340, Korea; 3 Department of Animal Science, South Dakota State University, Brookings, SD 57007, USA; 4 Department of Animal Science and Food Science and Human Nutrition, Michigan State University, East Lansing, MI 48824, USA

**Keywords:** Dietary Energy Density, Finishing, Growth, Hanwoo

## Abstract

**Objective:**

Our study aimed to investigate the effects of a 2% increase in dietary total digestible nutrients (TDN) value during the growing (7 to 12 mo of age) and fattening (13 to 30 mo of age) period of Hanwoo steers.

**Methods:**

Two hundred and twenty Hanwoo steers were assigned to one of two treatments: i) a control group (basal TDN, BTDN, n = 111 steers, growing = 70.5%, early fattening = 71.0%, late fattening = 74.0%) or high TDN (HTDN, n = 109 steers, growing = 72.6%, early = 73.1%, late = 76.2%). Growth performance, carcass traits, blood parameters, and gene expression of *longissimus dorsi* (*LD*) (7, 18, and 30 mo) were quantified.

**Results:**

Steers on the BTDN diets had increased (p≤0.02) DMI throughout the feeding trial compared to HTDN, but gain did not differ appreciably. A greater proportion of cattle in HTDN received Korean quality grade 1 (82%) or greater compared to BTDN (77%), while HTDN had a greater yield grade (29%) than BTDN (20%). Redness (a*) of *LD* muscle was improved (p = 0.021) in steers fed HTDN. Feeding the HTDN diet did not alter blood parameters. Steers fed HTDN diet increased (p = 0.015) the proportion of stearic acid and tended to alter linoleic acid. Overall, saturated, unsaturated, monounsaturated, and polyunsaturated fatty acids of *LD* muscle were not impacted by the HTDN treatment. A treatment by age interaction was noted for mRNA expression of myosin heavy chain (*MHC*) *IIA*, *IIX*, and stearoyl CoA desaturase (*SCD*) (p≤0.026). No treatment effect was detected on gene expression from *LD* muscle biopsies at 7, 18, and 30 mo of age; however, an age effect was detected for all variables measured (p≤0.001).

**Conclusion:**

Our results indicated that feeding HTDN diet could improve overall quality grade while minimum effects were noted in gene expression, blood parameters, and growing performance. Cattle performance prediction in the feedlot is a critical decision-making tool for optimal planning of cattle fattening and these data provide both benchmark physiological parameters and growth performance measures for Hanwoo cattle feeding enterprises.

## INTRODUCTION

In the last few decades, the Hanwoo (Korean native cattle) industry has been mainly driven by meat quality, primarily focusing on the proportion of intramuscular adipose tissue within the high-valued cuts of the loin and rib primals. In order to maximize intramuscular adipose tissue, several feeding strategies have been applied throughout various feeding stages. Feeding a high-energy diet for an extended period of time is the method that is routinely used in the Hanwoo feeding system of Korea. It has long been believed that high energy intake leads to increased fat content and body weight (BW) simultaneously [1].

Unlike other countries’ feeding systems (e.g., USA, Australia), the Hanwoo feeding program often stretches up to 30 to 32 mo following birth [2]. In 2017, 75.7% (291,676 head) of steers were harvested in Korea on and after 30 mo of age. Over 88% of these 30 mo or older cattle at harvest graded above a scale of 1 (1, 1+, and 1++), equivalent to or exceeding the USDA Prime grade (KAPE, 2021). The typical Korean feeding program provides high energy density diets in concentrate form and stimulates storing the exceeding energy as lipids as an intramuscular adipose tissue.

Since Hanwoo cattle are a later maturing breed, the extended feeding program needs to be used to maximize marbling content compared to other conventional beef breeds such as Angus, Shorthorn, or Charolais. However, prolonged fattening periods can cause increased hyperaccumulation of body fat, decreased feed efficiency, and lower muscle-to-bone cut-out yield [3]. Since feed cost is primary production cost, understanding the effect that an energy dense diet on animal growth and beef quality to establish an economically efficient feeding program while preventing overfeeding and excess feed being deposited as subcutaneous waste fat. We hypothesized that the offering a diet with an increased total digestible nutrients (TDN) conent may improve growth parameters and potentially shorten the feeding period. In addition, the higher energy diet may improve skeletal muscle growth by altering transcriptional regulation of muscle and adipose tissue. Therefore, the study was designed to demonstrate the effect of increased TDN value on animal growth, carcass traits, blood parameter, and *Longissimus dorsi* (LD) muscle gene expressions. Our study also provides benchmark data for a greater understanding of post-weaning growth patterns of Hanwoo beef steers throughout the growing, early, and late fattening periods.

## MATERIALS AND METHODS

### Animals and management

All experimental procedures were examined and permitted by the Institutional Animal Care and Use Committee of the National Institute of Animal Science (No. 2015-112). Two hundred and twenty steers (initial BW: 197.05±3.38 kg, 7 mo of age) were distributed into a BTDN group (n = 111) and HTDN group (n = 109). Steers were assigned into a pen (n = 4 steers/pen), resulting in twenty-eight pen replicates per treatment. The present study was categorized into the following periods: growing (7 to 12 mo of age), early fattening (13 to 20 mo of age), late fattening (21 to 30 mo of age). Commercial concentrate mix was provided in equal proportions twice daily (08:00 and 16:00) at a rate of 1.6% to 1.8% BW. Steers were alos offered *ad libitum* acces to hay during the growing period and 1 to 2 kg of rice straw was provided to each steer daily during both the early and late fattening period. Steers were fed treatment diets, formulated based on the Hanwoo feeding standard; TDN 69% to 70% for BW 200 to 350 kg, TDN 71% to 72% for BW 350 to 500 kg, and TDN 74% for BW>500 kg (wheres steers were programed to gain approximately 1 kg daily) [4].

The high TDN diet group (HTDN) was fed 3% greater TDN concentrate than the basal TDN diet group (BTDN) until the late fattening period where cattle were fed the same diet.

Performance analysis for the fattening period was conducted at the following intervals (total of 23 mo): growing, early fattening, and late fattening period of feeding. Steers were weighed every month during the experimental period on a scale (Newton HT-501A; CAS, Seoul, Korea) in the early morning before being fed. During the experimental feeding period, steers had *ad libitum* access to fresh water and a mineral block. The residual concentrate mix and forage were separately recorded daily to determine daily individual feed intake.

### Chemical analysis of diets

The chemical compositions of the diets were determined by collecting monthly feed samples and analyzing them according to AOAC methodology with 2-kg samples from each diet (AOAC, 2000). The diet formulations and chemical compositions are provided in Table 1.

### Serum constitution

Blood samples were collected at monthly intervals until cattle were harvested. Blood samples were drawn from the jugular vein of each steer with an 18-gauge needle into a 10-mL vacuum-sealed glass tube containing no additive (Becton Dickinson, Franklin Lakes, NJ, USA). Blood was allowed to clot for 24 h at 4°C and then centrifuged at 1,000×g for 20 minutes to obtain serum. Serum was stored at −70°C for subsequent analyses of non-esterified fatty acid (NEFA), albumin (ALB), glucose (GLU), triglyceride (TG), total protein (TP), and inorganic phosphorus (IP) levels. Blood metabolites were analyzed by an automated chemical analyzer (Automatic Analyzer, 7020; Hitachi Co., Ltd, Tokyo, Japan), following the manufacturer’s recommendation. All samples were run in triplicate determinations and considered for re-runs if the coefficient of variation among triplicate samples was greater than 10%.

### Carcass evaluation

All cattle (30 mo of age) were transported to a commercial abattoir (Pyeongchang, Gangwon Province, Korea). Carcasses were harvested according to humane procedures and were chilled for 24 h at 4°C and ribbed at the 13th rib. Personnel from the Korean Ministry for Food, Agriculture, Forestry, and Fisheries evaluated cold carcass weight, meat quality, and yield grade. Each carcass was evaluated for marbling score, rib fat thickness, ribeye area, meat color, fat color, and quality grade at the 13th rib. Yield grade and dressing percentage were calculated from the carcass data. *LD* muscles were removed from each carcass between the 12th and 13th rib, maintained at a temperature of 0°C to 5°C, and transferred to the University Meat Science Lab. The muscle samples for biochemical analysis were homogenized and stored in the freezer at −80°C. Samples were physicochemically analyzed for percent moisture, crude fat, crude protein, meat color, cooking loss, and shear force. The protein, fat, and moisture content (%) were analyzed using AOAC methodology (2000). Meat color was measured an average of 5 times for L* (lightness), a* (redness), and b* (yellowness) with a Chroma Meter (DR-10; Minolta Co., Tokyo, Japan).

Shear force was measured by cutting the muscle at a right angle to the direction of the muscle fiber in the form of a 3 cm thick steak, heating at 80°C for 40 minutes, and then allowing it to cool 20 minutes. A 1.27 cm diameter core was drilled cylindrical to the muscle fiber direction, and the shear force was measured using the Universal Testing Machine (Intron 4465; Instron, Wycombe, England).

Beef yield was based on the meat yield index (MYI) [5], where MYI = 68.184 – [0.625×rib fat thickness (mm)]+[0.130 ×rib eye area (cm^2^) – [0.024×carcass weight (kg)]+3.23 (A, MYI≥67.20; B, MYI between 63.30 and 67.20; C, MYI≤63.30).

### Fatty acid analysis

Total lipids were extracted from *LD* muscle collected from carcass via chloroform-methanol (2:1, v/v) extraction according to the procedure of Folch et al [6]. An aliquot of total lipid extract was methylated as described by Morrison and Smith [7]. The fatty acid composition was analyzed using gas chromatography (Varian 3600; Varian Med. Sys., Palo Alto, CA, USA), and for gas chromatography conditions, a silica capillary column (Omegawax 205, 30 m×0.32 mm ID, 0.25 μm film thickness) was used. The injection port temperature was 250°C, and the detector temperature was maintained at 260°C. The analysis result was calculated as a ratio (%) to the total peak area.

### *Longissimus dorsi* muscle biopsies

*LD* muscle samples were collected three times during the course of the experiment: i) 7 mo (feeding trial initiation), ii) 18 mo, and iii) 30 mo of age from 50 steers (BTDN, 24 steers; HTDN, 26 steers). Muscle samples (7 and 18-mo) were collected from *LD* muscle between the 13th rib and 1st lumber using a 6mm Bergstrom biopsy needle (Samyong, Daejeon, Korea) following the procedures described previously [8]. The *LD* samples were collected during animal harvest (30 mo of age). Collected samples (approximately 2 g) were placed in a asceptic medical container, then snap-frozen in liquid N_2_, and stored in a −80°C freezer until further processing.

### Gene expression

Total RNA was isolated from 1 g each of one *LD* muscle tissue (7, 18, and 30 mo) in 3 mL of ice-cold buffer containing TRI Reagent (Sigma-Aldrich, St. Louis, MO, USA). The total RNA concentration was determined based on the absorbance at 260 nm on an Epoch system (Synergy H1; BioTek Inc., Winooski, VT, USA). An acceptable range of 1.76 to 2.05 was used for the 260/280 ratio. One microgram of cDNA was synthesized from each sample, and real-time quantitative PCR was used to evaluate gene expression. Peroxisome proliferator-activated receptor gamma (*PPARγ*), stearoyl CoA desaturase (*SCD*), myogenin (*MyoG*), myosin heavy chain (*MHC*) *I*, *IIA*, and *IIX* mRNA expression were measured relative to an endogenous control, ribosomal protein S9 (*RPS9*). Measurements were performed in 96-well plates with 10 μL of TaqMan Universal PCR Master Mix (Applied Biosystems, Waltham, MA, USA), 4.5 μL of the appropriate forward and reverse primers (10 μM), 0.1 μL of the appropriate TaqMan probe, 2.0 μL of the cDNA sample, and 3.4 μL of nuclease-free water. The bovine primers, TaqMan probes, and accession numbers are presented in Table 2. Assays were performed on a 7500 Fast Real-Time PCR system (US/7500; Applied biosystems, USA) according to the thermal cycling parameters recommended by the manufacturer (40 cycles of 15 s at 95°C and 1 min at 60°C). Relative mRNA expression was quantified through the 2^−ΔΔCt^ method. All sample values were normalized against *RPS9* levels and expressed in arbitrary units.

### Statistical analysis

Growth performance data were analyzed by way of analysis of variance (SAS/ACCESS 9.4 Interface.; SAS Institute Inc, Cary, NC, USA) and the Duncan’s mean separation test for comparison of means was used. Repeated measures analysis of variance used Holm-Šídák multiple comparisons test for gene expression and blood parameter data (GraphPad Prism version 8.0.0 for Windows; GraphPad Software, San Diego, CA, USA). The fixed effects of treatment, age, and treatment ×age were determined for blood parameters, *LD* gene expression, and growth performance. Carcass characteristics, fatty acids compositions, and meat quality were analyzed by mixed analysis of variance. Mean separation was conducted using Tukey’s post hoc separation test by GraphPad for all variables.

The pen was considered as the experimental unit (n = 4 steers/pen) for dry matter intake (DMI), while steers served as experimental units for all other growth performance measures, carcass characteristics, meat quality, fatty acid composition, and gene expression data. An α level of 0.05 was used to determine significance, with tendencies discussed at p-values between 0.055 and 0.10. Treatment means were analyzed and separated with the least significant difference.

## RESULTS AND DISCUSSION

### Animal growth and blood parameters

The final BW of steers was not influenced (p<0.05) by dietary treatment. Average daily gain and gain to feed ratio were not altered by treatments throughout the feeding trial (Table 3). Throughout the experiment, steers that received the BTDN diet exhibited greater DMI in each period investigated (p<0.05). In the beef cattle industry, a grain-based, energy-dense diet is widely used during the finishing stage to improve the meat yield and marbling contents. However, increased TDN value in feed does not directly affect the overall animal growth, if cattle fed the lower TDN diet are able to consume extra feed to compensate for the decresed energy density of the diet. Our results are in agreement with a previous study that altered dietary energy (TDN 70% to 80%) and did not alter growth rate or final carcass weight in Angus× Chinese Xiangxi yellow cattle [9]. Fox et al [10] reported that DMI could decrease 2.7% as 1% of body fat is accumulated.

Despite similar gain of steers fed diets with different energy densities, steers offered BTDN had greater (p<0.05) DMI than HTDN steers. Numerous factors regulate appetite and the amount of ingested feed, factors such as sensory, habitual factors, circadian rhythm, and the energy status of steer can effect voluntary intake [11]. Among these factors, the energy status of animals is probably the primary modulator of feed intake, especially in the fattening periods of production. Friedman [12] suggested that DMI is decreased when the energy status is increased (greater adenosine-tri-phosphate: adenosin-di-phosphate [ATP:ADP] ratio), while feed intake is stimulated as energy status decreases (lesser ATP:ADP ratio). Therefore, the increased DMI in BTDN steers throughout the feeding trials was mainly due to the lower energy availability than than the HTDN diet and potentially differnces in whole body energy balance.

### Carcass traits

The mean carcass weight was not altered by dietary treatment (Table 4). The HTDN diet did not alter rib fat thickness, ribeye area, dressing percent, or marbling score. The steers that received HTDN showed a greater percentage (17%) of 1^++^ grade than BTDN (10%). Overall, 77% of BTDN steers had quality grades greater than grade 1 (1, 1^+^, and 1^++^), while 82% of HTDN cattle received quality grades greater than grade 1. The yield grade of steers fed BTDN and HTDN were 21(A):59(B):20(C) and 22(A):49(B):29(C), respectively.

In order to improve the marbling content of beef cuts, two strategies are mainly used. First, feeding a higher energy diet containing a higher starch to fiber ratio stimulates *de novo* fat synthesis and leads to increased fat accumulation in the carcass. The amount of lipogenesis from dietary fat source is minimal in the ruminant animal; propionate or GLU from grain diets are the primary substrate for fat synthesis [13]. Second, extending the feeding period to allow intramuscular fat to fully develop since intramuscular fat is the last adipose tissue depot to have complete lipid filling [14]. Typically, a Hanwoo steer feeding period often extends over 30 mo of age to maximize the marbling content in the harvested animal. The distinctive and genetically inherited trait of Hanwoo cattle allows them to accumulate relatively little rib fat despite having a greater marbling content. Hence, this allows for extending the feeding period for a longer period compared to other beef breeds such as Angus crossbred cattle that are commonly fed in Australia and the United States of America.

### Color

The lightness (L*) and yellowness (b*) of the LD muscle tended to be greater (p = 0.078, 0.071, respectively) in HTDN steers compared to BTDN steers (Table 4). The redness (a*) of the *LD* increased (p = 0.022) in HTDN. This suggests that feeding a high-energy diet can improve the luminosity of beef. Three factors, including muscle metabolism, intramuscular fat, and muscle fiber characteristics, alter the L* value [15]. Proximate composition analysis was conducted with *LD* muscle. Altering TDN of diet did not affect the moisture, crude fat, crude protein, and shear force measures.

### Blood metabolites

**Figure 1:**
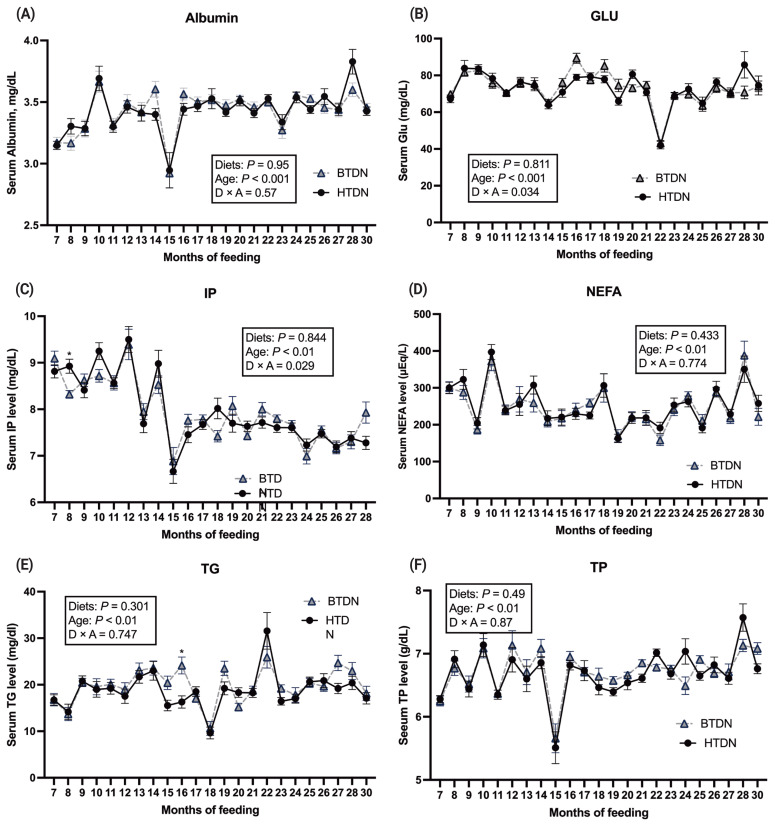
Sera metabolites response to the treatments: high TDN diet (HTDN); 3% increased TDN than BTDN, basal TDN diet (BTDN). (A) albumin (Alb, g/dL), (B) glucose (Glu, mg/dL), (C) inorganic phosphorus (IP, mg/dL), (D) non-esterified fatty acids (NEFA, μEq/L), (E) triglyceride (TG, mg/dL), (F) total protein (TP, g/dL).

No diet by age interaction was noted for ALB, NEFA, TG, or TP (Figure 1; panes A to F). An interaction was noted between age and diet for GLU (p = 0.034) and IP (p = 0.029). Albumin plays an important role in transporting numerous substances, especially NEFA, and often is used as a marker for monitoring the nutritional status of animals. For serum ALB, no treatment effect was noted, but there was an age effect (p<0.01). Serum ALB increased as animals aged. For serum NEFA, no treatment effect was noted, but there was an age effect (p<0.01). For serum TG, no treatment effect was noted, but there was an age effect (p<0.01). For serum TP, no treatment effect was found, but there was an age effect (p<0.01) noted. Serum GLU differed (p≤0.05) between treatments on 16 and 28 mo. The serum GLU level was in the range of previous studies with beef cattle fed high grain-based diets [16]. Serum IP differed (p≤0.05) between treatments on 8, 10, and 28 mo. Collectively, these data indicate that diet treatment only minimally influences the blood parameters status of Hanwoo cattle.

### mRNA gene expression

A treatment×age interaction was detected in *MHC IIA* (p = 0.026), *MHC IIX* (p = 0.013), and *SCD* (p = 0.013) (Table 5). The main effect of diet did not alter the expression of any other genes. The genes related to skeletal muscle fiber formation, including *MHC I*, *IIA*, *IIX*, were impacted (p<0.01) by the main effect of age. The Leas square means of *MyoG* was the greatest (p<0.01) at 18-mo, followed by 7 and 30-mo of age. The expression of *MHC I* was the greatest (p<0.01) at 18-mo of age, but *MHC IIA* was increased at both 7 and 8-mo of age. Experssion of *MHC IIX* was greatest (p<0.01) at 7-mo and diminished afterward. The expression of *PPARγ* and *SCD* were altered (p<0.01) by the main effect of age. At 30-mo of age, *PPARγ* was overexpressed compared to at a younger age. The expression of *SCD* was greater at 18-mo and the lowest at 7-mo. No treatment effects were detected for any *LD* muscle gene expression. The expression of *MyoG* was the greatest in 18 mo age followed by 7 and 30 mo of age. *MyoG* is one of the myogenic regulatory factors (*MRFs*) and plays an essential role in myogenic differentiation. Du et al [17] reported that most of the myogenic differentiation occurs prenatally, and adult muscle myogenesis is related to satellite cell activation. Satellite cells are single cell adult muscle precursor cells located under the basal lamina of the muscle fiber. Satellite cells contribute myonuclei to preexisting muscle fibers and play a critical role in muscle hypertrophy and regeneration. Overexpression *MyoG* in 18 mo of age may indicate enhanced satellite cell activity. It was previously reported that the proportion of satellite cells and their mitotic activity gradually declined with age [18]. Our data also showed that *MyoG* expression was decreased in 30 mo old steers.

Age effects were also noted for the expression of *MHCs*. *MHC I*, *IIA*, and *IIX* are predominant miscle tyoe isoforms in adult bovine skeletal muscle. Type I, slow-twitch muscle fibers that use oxidative metabolism, mainly express *MHC I* isoform [19]. The expression of *MHC I* was the greatest in 18 mo of age and the lowest at 30 mo of age. In 18 mo, steers fed HTDN overexpressed (p<0.05) *MHC I* compared to BTDN (Table 6). *MHC IIA*, is intermediate between *MHC I* and *IIX*, and was also expressed the most (p<0.01) at 7 and 18 mo of age. Amongst muscle fiber types, type I muscle fibers positively correlated with the marbling content of beef cuts and store more lipid as a fuel source of oxidative metabolism compared to muscles that use glycolytic metabolism [20]. The expression of *MHC IIX* was increased (p<0.05) by the HTDN diet at 18 mo of age (Table 6), but the main effect for age was greatest during the growing stage (7 mo) of age (p<0.01). *MHC IIX* is the *MHC* isoform that is fast-twitched, has glycolytic muscle fibers, and prefers carbohydrates as a fuel source compared to oxidative muscle fiber types. As steers age, the glycolytic-dominant metabolism of the *LD* muscle might shift towards oxidative metabolism following the increased availability of substrate needed for fuel, for instance, intramuscular adipose tissue accumulation.

Peroxisome proliferator-activated receptors (*PPAR*), a nuclear hormone receptor superfamily member, play a significant role in various types of cell metabolism. Amongst *PPAR* isoforms, *PPARγ* is the key transcription factor during adipogenic differentiation and plays a critical role in controlling lipid metabolism [21]. In our current study, treatment effects were not detected while age effects were noted (p<0.01). As discussed earlier, intramuscular fat is developed in the later stages of animal growth, increased *PPARγ* expression can indicate that high adipogenic activity is occurring around 30 mo of age. Enzyme activity and *SCD* gene expression in bovine adipose tissues were evaluated to investigate fat softness and meat quality [13]. The overexpression of *SCD* can lead to the increased proportion of unsaturated fatty acids (UFAs) in beef fat by catalyzing the synthesis of saturated fatty acids (SFA) such as stearic acid (C18:0), palmitic acid (C16:0) to monounsaturated fatty acids (MUFA) such as oleic acid (C18:1), and palmitoleic acid (C16:1) [22]. Increased MUFA in beef cuts can improve the overall liking and has a moderate relationship to tenderness in both beef and lamb [23]. In the present study, the *SCD* expression seems to peak (p<0.01) during the early fattening period (18 mo), and this finding is partly in agreement with the previous research. Martin et al [24] reported that the *SCD* gene expression of subcutaneous fat was peaked at 12 mo of age and diminished as animals aged.

### Fatty acids in *longissimus dorsi* muscle

The *LD* composition of palmitic acid, oleic acid, linoleic acid, and arachidonic acid was not impacted by the HTDN diet (Table 7). Steers fed HTDN diet increased proportion of stearic acid (p = 0.015) and a tendency was detected for linoleic acid (p = 0.082). Overall, SFA, UFA, MUFA, and polyunsaturated fatty acids (PUFA) content of the *LD* muscle were not impacted by TDN treatment.

Fatty acid composition is one of the most critical determinants of beef quality and a desribale eating experience for beef consumers. It has previously been reported that the fatty acid composition of Hanwoo cattle is unique compared to American Angus-influence or Australian crossbred cattle, and this is likely due to differences in fatty acid composition. Lee et al [25] reported that Hanwoo beef contains a greater concentration of oleic acids ranging from 53% to 56% and a greater proportion of MUFA/SFA while maintaining a lower PUFA/SFA. In the current study, steers received HTDN increased (p = 0.015) stearic acid (C18:0) content in the *LD* compared to BTDN steers. However, treatment effects on percent fatty acids were not appreciable. Stearic acid content of the *LD* ranges from 12.9% to 16% in Charolais steers [26], 13.4% and 13.6% in Angus and Brahman cattle, respectively [27], and 19% in most conventionally reared U.S. beef [28]. While stearic acid differs among breed types, stearic acid composition usually ranges from 13% to 19%. Our data showed a lower (11.23% to 11.67%) concentration of stearic acids than demonstrated in previous studies with other beef breeds. It seemed that Hanwoo cattle contain a relatively lower stearic acid proportion than other breeds at the point of harvest. This finding is in agreement with the data presented by Lee et al [29]. The extended feeding period of Hanwoo cattle is probably the primary factor that changes the fatty acid composition. Smith et al [27] stated that three significant factors affect the fatty acid composition of beed: i) the age of the beef animal, ii) diet energy density, and iii) breed type. This study also pointed out that age and breed type of beef cattle can alter the concentration of MUFA by regulating *SCD* activity in the *LD*. Our data also indicated that *SCD* gene expression of loin muscle peaked after 18 mo of age

Linolenic acid (C18:3n3), one of the essential fatty acids in human nutrition, tended to be increased (p = 0.082) in HTDN steer, but the percentage reposnse was not appreciable. Linolenic acid composition is usually around 0.7% in Angus, and Brahman cattle and can be increased by approximately 20% by offering a rich source of linolenic acid such as flaxseed [30]. Although the statistical differences were detected in the percentage of stearic acid and linoleic acid, the actual fatty acid alteration appears minimal.

## CONCLUSION

The present study measured the effects of providing a diet with 2% increased TDN value to Hanwoo steers from 7 to 30 mo of age. Providing a greater TDN diet did not alter the growth performance and had minimal effects on feed intake. It also noted that blood parameters and expression of most myogenic and adipogenic genes were not altered by dietary treatments, while *MHC IIA* and *IIX* were increased at 18 mo of age (early fattening) in steers fed HTDN. Steers fed HTDN diet also showed enhanced quality grade compared to steers fed BTDN.

## Figures and Tables

**Table 1:** Ingredient formulation and analyzed chemical compositions of concentrate mix and forage

Item^1),2)^	Growing		Early fattening		Late fattening		Timothy	Rice straw
		
	BTDN	HTDN	BTDN	HTDN	BTDN	HTDN		
Ingredient (% DM)								
Corn	20	22	25	26	29	30.5	-	-
Barley	-	-	-	-	8	8	-	-
Cottonseed whole	-	-	-	-	3	3	-	-
Wheat	18	18	19	19	19	19.5	-	-
Wheat bran	14	12	13	10	3	2	-	-
Rice bran	2	2	5	5	4	2	-	-
Soy hull	-	-	1	-	2	-	-	-
Corn gluten meal	7	7	9	11	10	11	-	-
Coconut oil	8	6	7	7	5	3	-	-
Palm kernel meal	8	8	7	7	5	5	-	-
Canola meal	5	5	2	3	1.5	3	-	-
Soybean meal	3	5	-	-	-	-	-	-
DDGS	4	4	2	2	2	2.5	-	-
Molasses cane	3	3	3	3	3	3	-	-
Limestone	3	3	3	3	2	2	-	-
Salt	0.8	0.8	0.8	0.8	0.8	0.8	-	-
Sodium bicarbonate	0.4	0.4	0.5	0.5	0.7	0.7	-	-
Magnesium oxide	0.3	0.3	0.3	0.3	0.3	0.3	-	-
Mineral premix	0.2	0.2	0.22	0.22	0.22	0.22	-	-
Vitamin premix	0.18	0.18	0.05	0.05	0.15	0.15	-	-
Others	3.12	3.12	2.13	2.13	1.33	1.33	-	-
Total	100	100	100	100	100	100	-	-
Chemical composition								
DM	87.87	87.69	87.79	87.94	87.43	87.57	90.38	90.68
CP	16.00	16.48	13.00	13.39	12.38	12.75	6.58	2.70
EE	3.42	3.76	3.74	4.57	4.11	5.04	2.92	1.98
Crude fiber	6.74	6.50	6.76	6.36	6.73	5.85	30.47	34.57
Crude ash	9.12	8.48	9.04	8.62	7.51	7.09	4.55	11.46
Calcium^2)^	1.25	1.20	1.25	1.23	0.89	0.81	0.22	0.13
Phosphorus^2)^	0.57	0.56	0.57	0.56	0.47	0.45	0.22	0.05
ADF	11.36	10.84	10.84	10.35	10.12	8.82	33.26	39.15
NDF	28.25	27.10	27.61	26.85	25.34	23.63	58.59	67.82
TDN	70.53	72.65	71.00	73.13	74.00	76.22	-	-

TDN, total digestible nutrients; BTDN, basal TDN; HTDN, high TDN; DDGS, Distiller’s dried grains with soluble; DM, dry matter; CP, crude protein; EE, ether extract; CA, crude ash; ADF, acid detergent fiber; NDF, neutral detergent fiber.

1) All values except DM on a dry matter basis.

2) Ca and P values were analyzed by the manufacturer.

**Table 2:** Primer nucleotide sequence for gene expression

Genes	Accession No.	Sequence (5′ to 3′)
*MyoG*		
Forward		AGAAGGTGAATGAAGCCTTCGA
Reverse		GCAGGCGCTCTATGTACTGGAT
Probe	AF091714	6FAM-CCCAACCAGAGGCTGCCCAAAGT-TAMRA
*MHC I*		
Forward		CAGCTCCAGAAGATCGACAAATC
Reverse		CTGCTCCACTTGACTGACGTTT
Probe	AB059400	6FAM-AGGGCCGCTTCCATGCCC-TAMRA
*MHC IIA*		
Forward		CCCCGCCCCACATCTT
Reverse		TCTCCGGTGATCAGGATTGAC
Probe	AF091714	6FAM-TCTCTGACAACGCCTATCAGTTCAT-TAMRA
*MHC IIX*		
Forward		GGCCCACTTCTCCCTCATTC
Reverse		CCGACCACCGTCTCATTCA
Probe	AB059399	6FAM-CGGGCACTGTGGACTACAACATTACT-TAMRA
*PPARγ*		
Forward		ATCTGCTGCAAGCCTTGGA
Reverse		TGGAGCAGCTTGGCAAAGA
Probe	NM181024	6FAM-CTGAACCACCCCGAGTCCTCCCAG-TAMRA
*SCD*		
Forward		TGCCCACCACAAGTTTTCAG
Reverse		GCCAACCCACGTGAGAGAAG
Probe	AB075020	6FAM-CCGACCCCCACAATTCCCG-TAMRA

*MyoG*, myogenin; *MHC*, myosin heavy chain; *PPARγ*, peroxisome proliferator-activated receptor gamma; *SCD*, stearoyl CoA desaturase.

**Table 3:** Growth performance of steers fed with different TDN diets

Items	BTDN	HTDN	SEM	p-value
Initial BW (kg)	196.90	197.19	0.14	0.967
Final BW (kg)	697.80	697.98	0.09	0.989
ADG (kg)				
Growing^1)^	0.67	0.72	0.02	0.149
Early fattening^1)^	0.83	0.85	0.01	0.562
Late fattening^1)^	0.79	0.81	0.01	0.678
Overall^1)^	0.78	0.80	0.01	0.208
DMI (kg)^2)^				
Growing^1)^	6.87	6.82	0.03	0.023
Early fattening^1)^	8.98	8.91	0.04	0.001
Late fattening^1)^	9.64	9.41	0.12	0.001
Overall^1)^	9.13	8.96	0.08	0.001
G:F				
Growth^1)^	0.13	0.12	0.00	0.568
Early^1)^	0.08	0.08	0.00	0.765
Late^1)^	0.09	0.09	0.00	0.676
Overall^1)^	0.10	0.09	0.00	0.934
FCR				
Growth^1)^	8.58	9.84	0.63	0.351
Early^1)^	12.89	12.14	0.38	0.503
Late^1)^	11.85	11.75	0.05	0.917
Overall^1)^	11.42	11.42	0.00	0.998

TDN, total digestible nutrients; BTDN, basal TDN; HTDN, high TDN; SEM, standard error of the mean; BW, body weight; ADG, average daily gain; DMI, dry matter intake; FCR, feed conversion ratio.

1) Growing, 7 to 12 mo of age; early fattening, 13 to 20 mo of age; late fattening, 21 to 30 mo of age; overall, the mean of 7 to 30 mo.

2) DMI = (concentrate feed intake×dry matter %)+(forage intake×dry matter %).

**Table 4:** Carcass traits responses to treatments

Items	BTDN	HTDN	SEM	p-value
Carcass weight (kg)	428.86	434.83	2.98	0.367
Back-fat thickness (mm)	12.32	12.34	0.01	0.966
Rib-eye area (cm^2^)	88.19	89.23	0.52	0.433
Dressing (%)	64.91	64.83	0.04	0.854
Marbling score	5.33	5.42	0.04	0.746
Quality grade^1)^				
(1^++^:1^+^:1:2)	10:43:24:23	17:35:30:18	-	-
Yield grade^2)^				
(A:B:C)	21:59:20	22:49:29	-	-
CIE L*	38.48	39.14	0.33	0.078
a*	21.74	22.43	0.35	0.022
b*	9.78	10.17	0.20	0.071
Moisture (%)	62.89	62.77	0.06	0.851
Crude fat (%)	15.10	15.48	0.19	0.643
Crude protein (%)	20.46	20.12	0.17	0.180
Shear force (kg/1.27 cm, core diameter)	3.85	3.76	0.04	0.571

TDN, total digestible nutrients; BTDN, basal TDN; HTDN, high TDN; SEM, standard error of the mean; CIE, International Commission on Illumination.

1) Korean Hanwoo beef quality grading: beef is qualified by 5 grades, 1^++^, 1^+^, 1, 2, and 3, depending on the degree of marbling, meat, and fat color, firmness of loin muscle between 13th rib and the 1st lumbar vertebra.

2) Yield grading consists of three grades, A, B, and C, depending on the meat yield index value. The equation is following; 68.184 – [0.625×rib fat thickness (mm)] + [0.130×rib eye area (cm^2^) – [0.024×carcass weight (kg)]+3.23 (A: MYI ≥67.20; B: MYI between 63.30 and 67.20; and C: MYI≤63.30). All chemical analyses (moisture, crude fat, crude protein, and shear force) were conducted using LD at the 12th–13th rib.

**Table 5:** Effect of TDN-adjusted diets and age on the least-square means of *longissimus dorsi* muscle mRNA gene expression (arbitrary nits)

Genes^1)^	Diets^2)^		SEM^3)^	Age^4)^			SEM^5)^	p-value		
		
	BTDN	HTDN		7	18	30		Treatments	Age	Trt×age
*MyoG*	0.83	0.93	0.108	0.85^b^	1.17^a^	0.62^c^	0.152	0.292	<0.001	0.208
*MHC I*	0.84	0.97	0.146	0.77^ab^	1.26^a^	0.69^b^	0.104	0.122	<0.001	0.142
*MHC IIA*	1.03	1.04	0.172	1.35^a^	1.26^a^	0.49^b^	0.121	0.863	<0.001	0.026
*MHC IIX*	0.77	0.87	0.138	0.74^a^	0.14^b^	0.33^b^	0.098	0.234	<0.001	0.013
*PPARγ*	2.29	2.59	0.441	1.58^b^	1.89^b^	3.84^a^	0.312	0.243	<0.001	0.225
*SCD*	2.70	2.81	0.396	1.09^b^	3.89^a^	2.70^ab^	0.629	0.796	<0.001	0.013

TDN, total digestible nutrients.

1) mRNA gene expression of the; *MyoG*, myogenin; *MHC*, myosin heavy chain I, IIA, and IIX; *PPARγ*, peroxisome proliferator-activated receptor gamma; *SCD*, stearoyl-CoA desaturase; as an endogenous control.

2) Main effect of treatments: HTDN, high TDN diet, 3% increased TDN than BTDN; BTDN, basal TDN diet.

3) Standard error of the mean for the main effect of treatment.

4) Within a row, least-square means without a common superscript duffer (p<0.05) due to age.

5) Standard error of the mean for the main effect of age.

**Table 6:** Treatment effects of TDN-adjusted diet of *longissimus dorsi* muscle mRNA gene expression

Genes^1)^	7 mo				18 mo				30 mo			
		
	BTDN^2)^	HTDN^2)^	SEM^3)^	p-value	BTDN^2)^	HTDN^2)^	SEM^3)^	p-value	BTDN^2)^	HTDN^2)^	SEM^3)^	p-value
Myogenin	0.91	0.79	0.159	0.830	1.08	1.25	0.149	0.588	0.50	0.74	0.155	0.300
*MHC I*	0.82	0.73	0.151	0.905	1.11	1.42	0.140	0.076	0.60	0.78	0.148	0.573
*MHC IIA*	1.53	1.17	0.218	0.297	1.49^b^	2.00^a^	0.203	0.038	0.45	0.53	0.211	0.967
*MHC IIX*	0.80	0.68	0.142	0.765	1.17^b^	1.60^a^	0.133	0.005	0.34	0.33	0.133	1.000
*PPARγ*	1.15	2.02	0.440	0.143	1.75	2.02	0.415	0.888	3.96	3.72	0.469	0.939
*SCD*	1.09	1.10	0.621	0.999	3.12^b^	4.48^a^	0.607	0.077	3.89	2.85	0.658	0.312

TDN, total digestible nutrients.

1) mRNA gene expression of the; *MyoG*, myogenin; *MHC I, IIA*, and *IIX*, myosin heavy chain I, IIA, and IIX; *PPARγ*, peroxisome proliferator-activated receptor gamma; *SCD*, stearoyl-CoA desaturase.

2) Main effect of treatments: HTDN, high TDN diet, 3% increased TDN than BTDN; BTDN, basal TDN diet.

3) Standard error of the mean for the main effect of treatment.

a,b Within a row, least-square means without a common superscript differ (p<0.05) due to treatments.

**Table 7:** The proportion (% of total fatty acids) of fatty acids in *longissimus dorsi*

Fatty acids	BTDN	HTDN	SEM	p-value
Myristic acid (C14:0)	3.40	3.27	0.06	0.129
Palmitic acid (C16:0)	27.83	27.66	0.09	0.484
Palmitoleic acid (C16:1n7)	4.58	4.47	0.06	0.274
Stearic acid (C18:0)	11.23^*^	11.67^*^	0.22	0.015
Oleic acid (C18:1n9)	50.08	50.00	0.04	0.829
Vaccenic acid (C18:1n7)	0.52	0.51	0.00	0.940
Linoleic acid (C18:2n6)	1.94	2.01	0.04	0.241
γ-Linoleic acid (C18:3n6)	0.04	0.04	0.00	0.949
Linolenic acid (C18:3n3)	0.08	0.08	0.00	0.082
Eicosenoic acid (C20:1n9)	0.48	0.46	0.01	0.404
Arachidonic acid (C20:4n6)	0.18	0.17	0.00	0.496
SFA^1)^	42.46	42.60	0.07	0.682
UFA^1)^	57.54	57.40	0.07	0.682
MUFA^1)^	55.30	55.09	0.10	0.535
PUFA^1)^	2.24	2.31	0.04	0.305

TDN, total digestible nutrients; BTDN, basal TDN; HTDN, high TDN; SEM, standard error of the mean.

1) SFA, saturated fatty acid; UFA, unsaturated fatty acids; MUFA, monounsaturated fatty acids; PUFA, polyunsaturated fatty acids.

* Means between treatments (BTDN and HTDN) is significantly different p<0.05.
